# Supplementary material for: In Silico Identification of Potential Natural Product Inhibitors of Human Proteases Key to SARS-CoV-2 Infection
Source: Molecules. 2020 Aug 22;25(17):3822. doi: 10.3390/molecules25173822 (PMC7504347; doi:10.3390/molecules25173822)
Supplement: Supplementary file 1 [file molecules-25-03822-s001.zip › SIFigures_VivekAnanth_Molecules.pdf]

Supplementary Figures S1-S7

for

In silico identification of potential natural product inhibitors of human proteases key to SARS-CoV-2 infection

R.P. Vivek-Ananth[1,2], Abhijit Rana[2,3], Nithin Rajan[1], Himansu S. Biswal[2,3,*,] Areejit Samal[1,2,*,$]

[1]*The Institute of Mathematical Sciences (IMSc), Chennai 600113, India*

[2]*Homi Bhabha National Institute (HBNI), Mumbai 400094, India*

[3]*School of Chemical Sciences, National Institute of Science Education and Research (NISER), Bhubaneswar 752050, India*

[*]*Correspondence to: asamal@imsc.res.in or himansu@niser.ac.in*

[$]Lead Contact

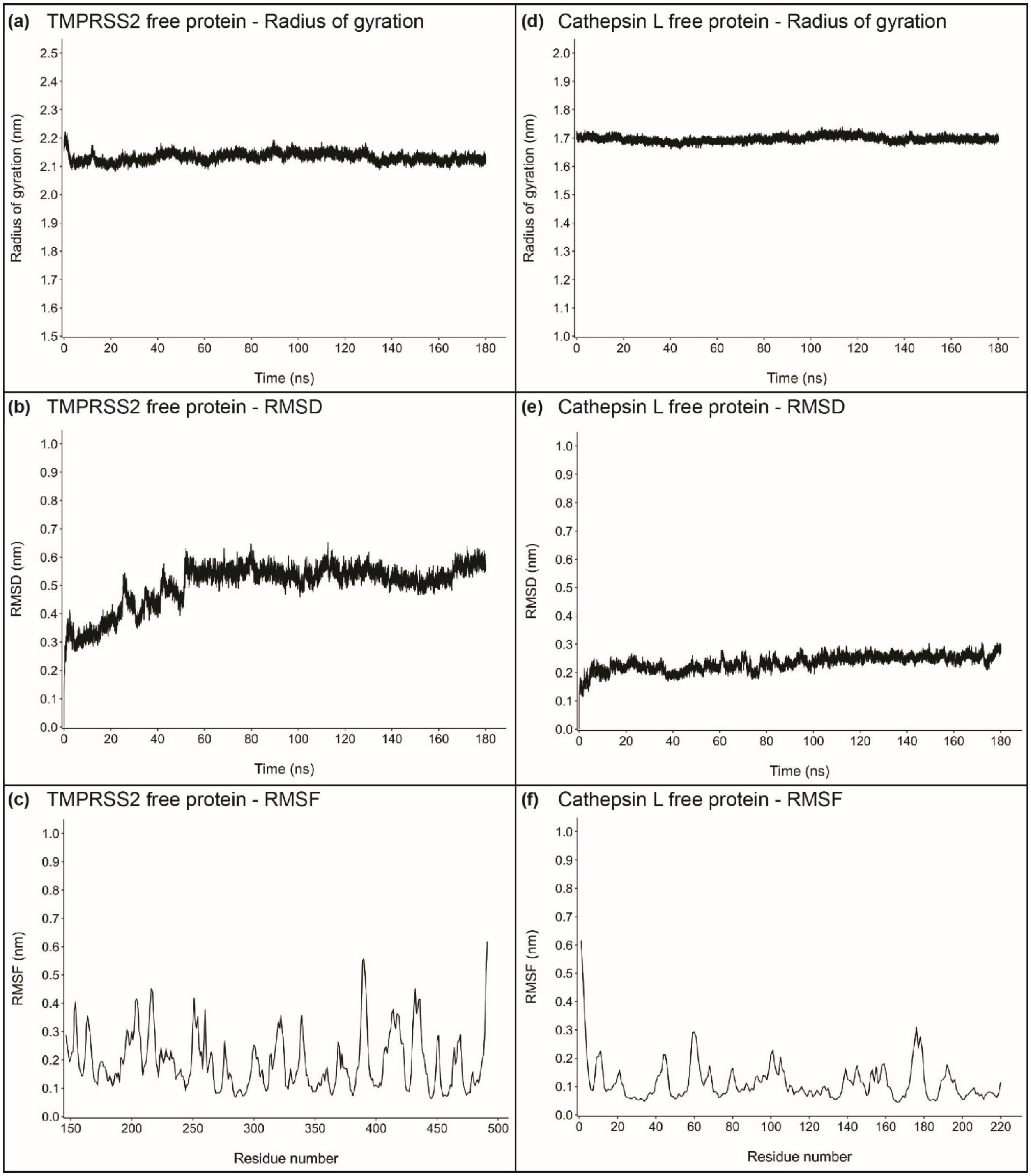

Figure S1: (a) Radius of gyration, (b) RMSD, and (c) RMSF for TMPRSS2 free protein. (d) Radius of gyration, (e) RMSD, and (f) RMSF for cathepsin L free protein.

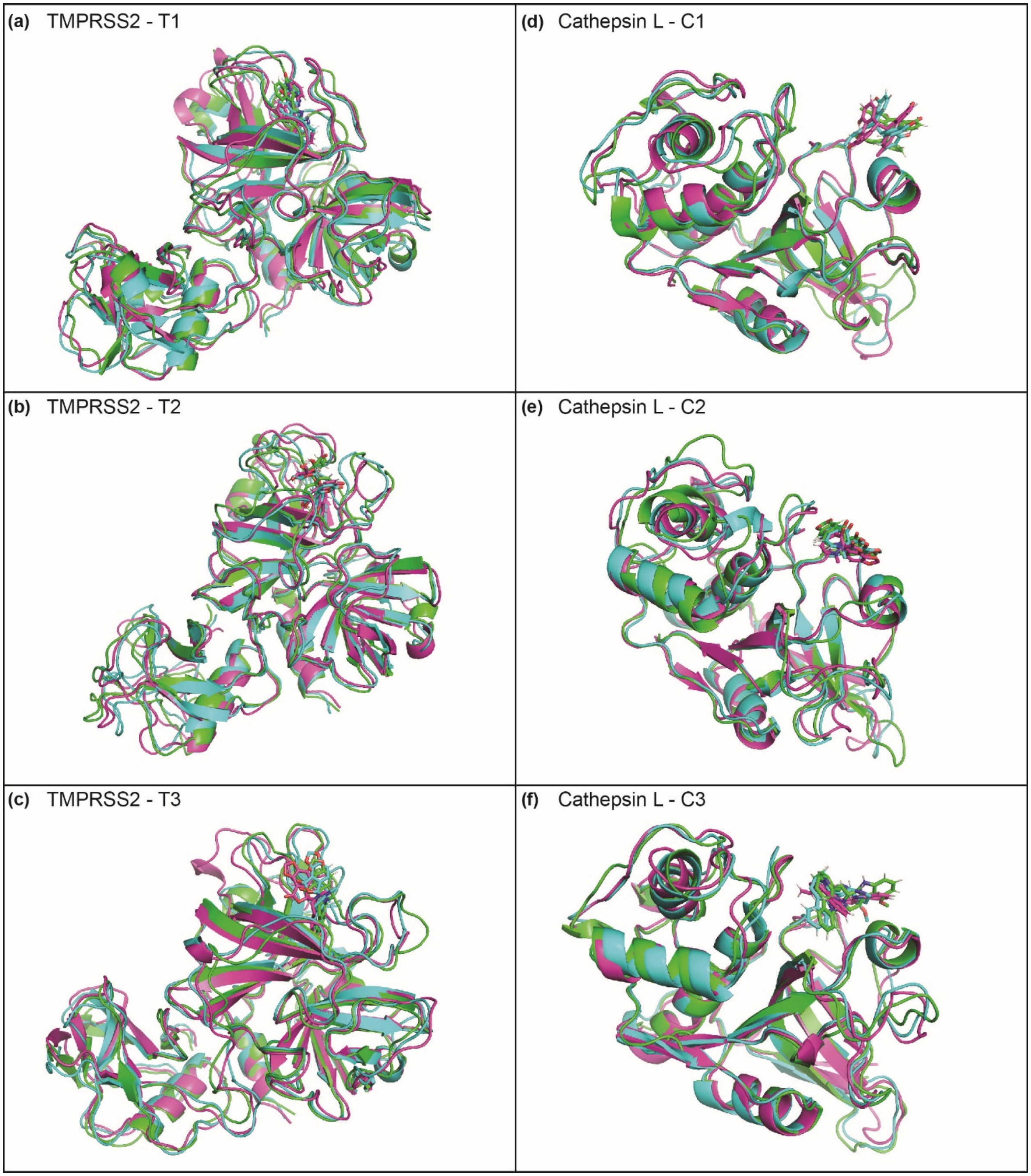

Figure S2: Superimposition of the snapshots at 120 ns, 140 ns and 160 ns of (a) TMPRSS2-T1 complex, (b) TMPRSS2-T2 complex, (c) TMPRSS2-T3 complex, (d) cathepsin L-C1 complex, (e) cathepsin L-C2 complex and (f) cathepsin L-C3 complex obtained from their respective MD simulation trajectories.

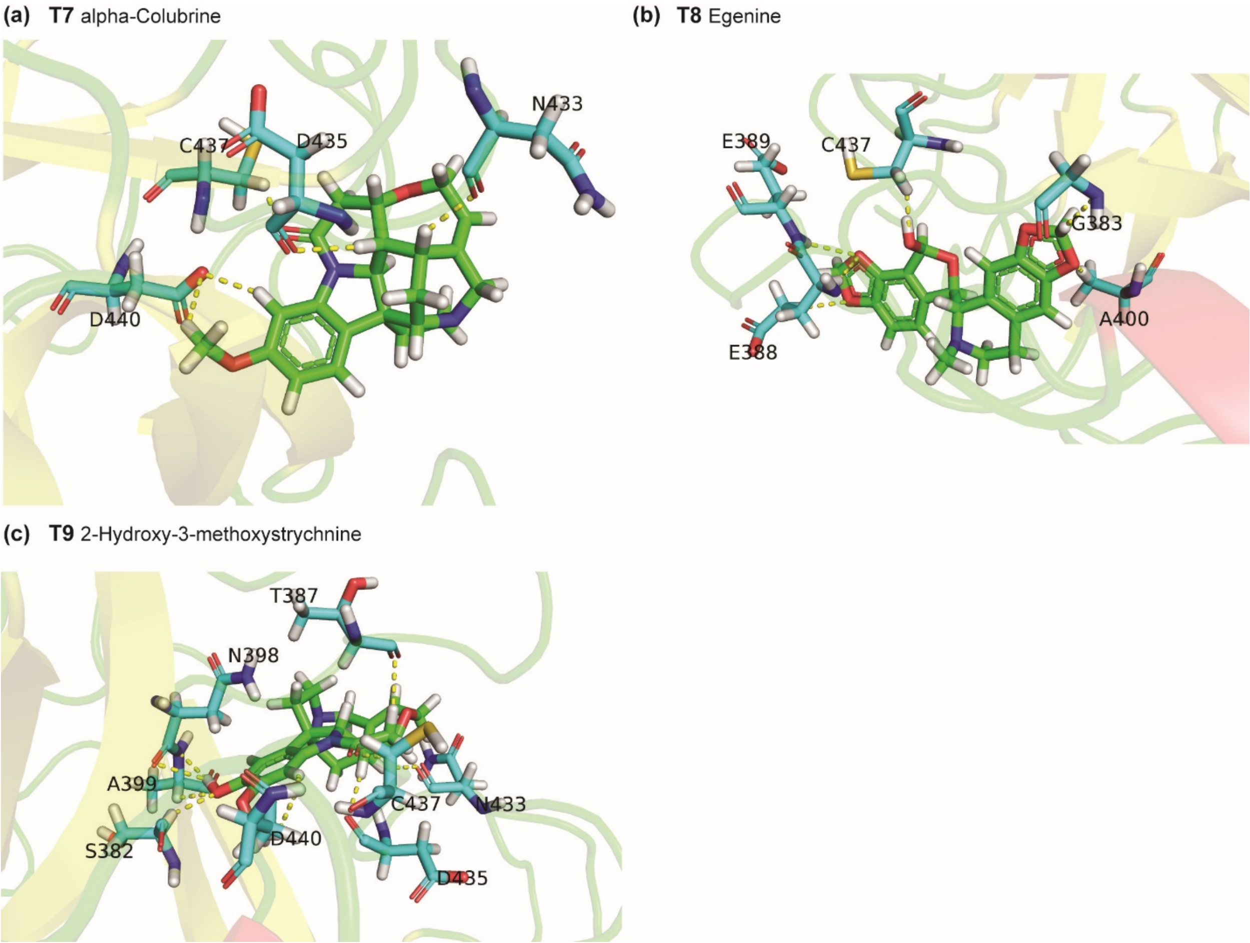

Figure S3: Cartoon representation of the protein-ligand interactions of the phytochemical inhibitors of TMPRSS2. Interactions of TMPRSS2 residues with atoms of (a) T7, (b) T8, and (c) T9. The carbon atoms of the ligand are shown in green colour while the carbon atoms of the amino acid residues in TMPRSS2 are shown in cyan colour. TMPRSS2 residues interacting with the ligand atoms via hydrogen bonds or $\boldsymbol{\pi}$-$\boldsymbol{\pi}$ stacking are labelled with the corresponding single letter residue code along with their position in the protein sequence. The hydrogen bonds and $\boldsymbol{\pi}$-$\boldsymbol{\pi}$ stacking are displayed using yellow and red dotted lines, respectively.

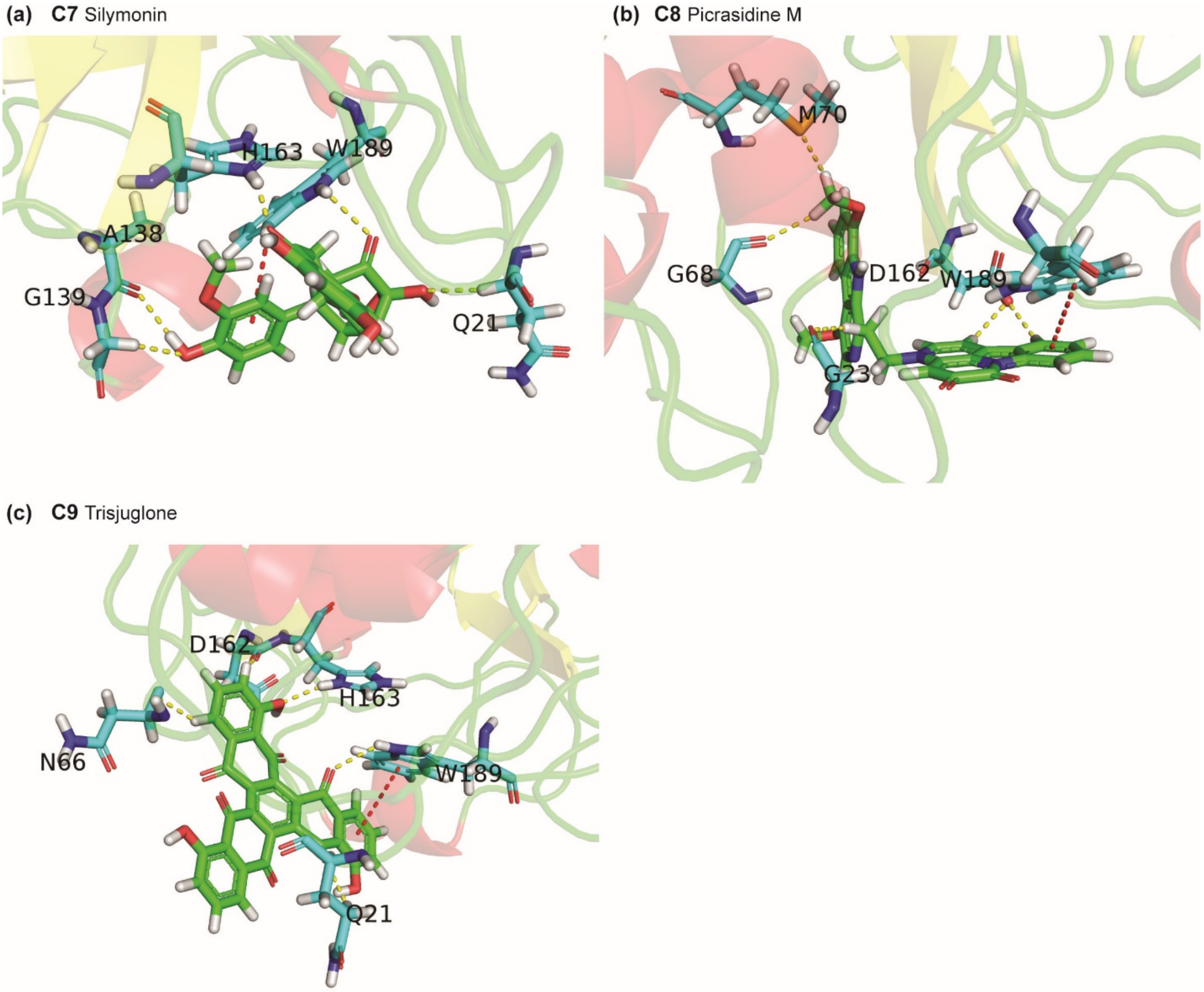

Figure S4: Cartoon representation of the protein-ligand interactions of the phytochemical inhibitors of cathepsin L. Interactions of cathepsin L residues with atoms of (a) C7, (b) C8, and (c) C9. The carbon atoms of the ligand are shown in green colour while the carbon atoms of the amino acid residues in cathepsin L are shown in cyan colour. Cathepsin L residues interacting with the ligand atoms via hydrogen bonds or ***π-π*** stacking are labelled with the corresponding single letter residue code along with their position in the protein sequence. The hydrogen bonds and ***π-π*** stacking are displayed using yellow and red dotted lines, respectively.

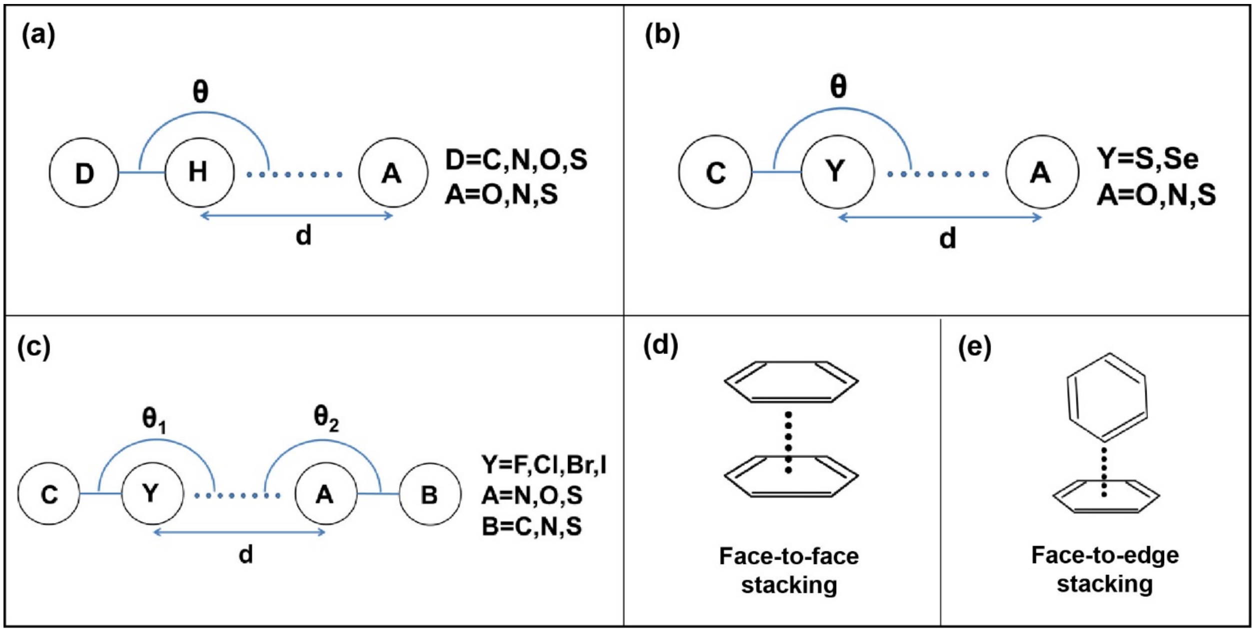

Figure S5: Geometric criteria for the identification of protein-ligand interactions. (a) Hydrogen bond, (b) Chalcogen bond, (c) Halogen bond, (d) face-to-face π-π stacking, and (e) face-to-edge π-π stacking.

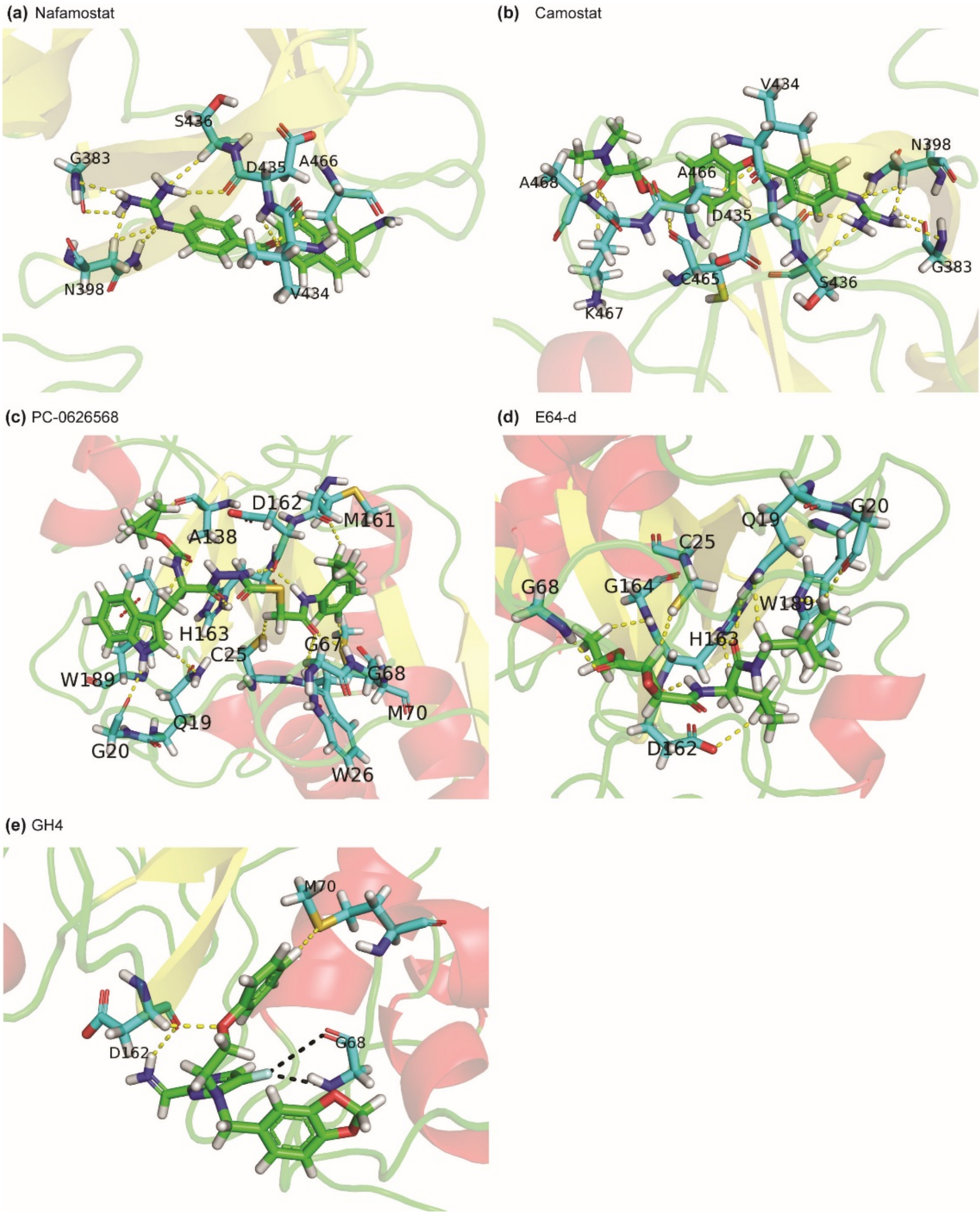

Figure S6: Cartoon representation of the protein-ligand interactions of the known inhibitors of TMPRSS2 and cathepsin L. Interactions of TMPRSS2 residues with atoms of (a) Nafamostat, and (b) Camostat. Interactions of cathepsin L residues with atoms of (c) PC-0626568, (d) E-64d and (e) GH4. The carbon atoms of the ligand are shown in green colour while the carbon atoms of the amino acid residues in TMPRSS2 or cathepsin L are shown in cyan colour. TMPRSS2 or cathepsin L residues interacting with the ligand atoms via hydrogen bonds or ***π-π*** stacking or halogen bonds are labelled with the corresponding single letter residue code along with their position in the protein sequence. The hydrogen bonds, ***π-π*** stacking and halogen bonds are displayed using yellow, red and black dotted lines, respectively.

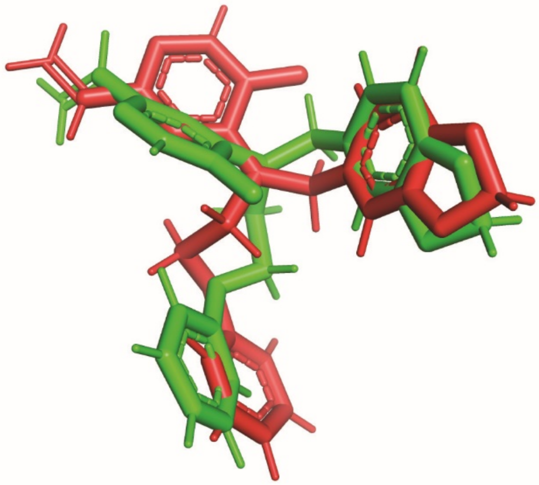

Figure S7: Superimposition of the docked pose of GH4 with cathepsin L obtained from AutoDock Vina (shown in green colour) and the pose of GH4 in the co-crystallized structure with cathepsin L (shown in red colour).
